# Recent Advances in Nanostructured Biomimetic Dry Adhesives

**DOI:** 10.3389/fbioe.2013.00022

**Published:** 2013-12-30

**Authors:** Andras Pattantyus-Abraham, Jeffrey Krahn, Carlo Menon

**Affiliations:** ^1^MENRVA Research Group, School of Engineering Science, Simon Fraser University, Burnaby, BC, Canada

**Keywords:** biomimetic, dry adhesive, nanostructure, mold, gecko adhesive

## Abstract

The relatively large size of the gecko and its ability to climb a multitude of structures with ease has often been cited as the inspiration upon which the field of dry adhesives is based. Since 2010, there have been many advances in the field of dry adhesives with much of the new research focusing on developing nanoscale and hierarchical features in a concentrated effort to develop synthetic gecko-like dry adhesives which are strong, durable, and self-cleaning. A brief overview of the geckos and the hairs which it uses to adhere to many different surfaces is provided before delving into the current methods and materials used to fabricate synthetic gecko hairs. A summary of the recently published literature on bio-inspired, nanostructured dry adhesives is presented with an emphasis being placed on fabrication techniques.

## Introduction

The field of biomimetic dry adhesives has flourished in recent years. From the earliest report on synthetic, gecko-inspired, adhesives (Geim et al., 2003; Sitti and Fearing, 2003), there has been substantial progress in understanding the theory, materials, and fabrication processes underlying dry adhesion. The optimization of adhesive properties involves the synergistic exploration of solid mechanics, materials synthesis and properties, interface properties, micro- and nanostructuring, all supported by the high level of function observed in biological examples.

Interest in biomimetic dry adhesives has been driven, in many cases, by the desire to achieve climbing robots and has been inspired largely by observation of the Tokay gecko which may weigh up to 300 g and reach lengths up to 35 cm – approximately the same size and length as many climbing robots using synthetic dry adhesives as an attachment method (Daltorio et al., 2006; Kim et al., 2008; Krahn et al., 2011). The adhesion strength produced by the gecko foot pad can reach 10 N for a 100 mm^2^ (Autumn et al., 2000) area and is used on a wide variety of surfaces providing a yet unachieved goal for synthetic dry adhesion strength and utility.

**Figure 1:**
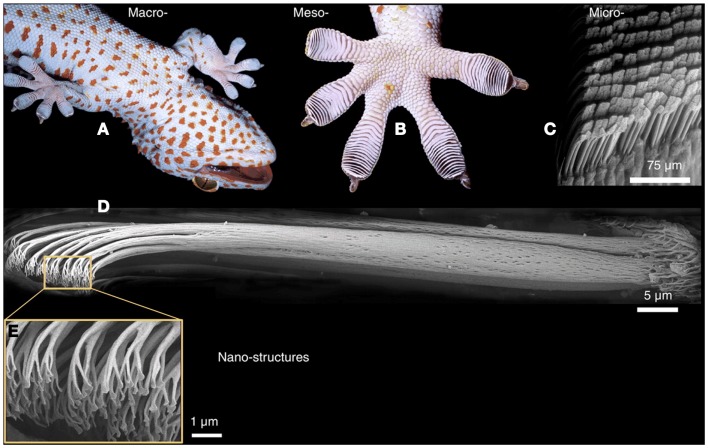
**Structural hierarchy of the gecko adhesive system**. **(A,B)** Gecko toe pads. **(C)** Gecko setae. **(D)** A single seta of the tokay gecko. **(E)** Gecko spatulae. From Autumn et al. (2006a).

**Figure 2:**
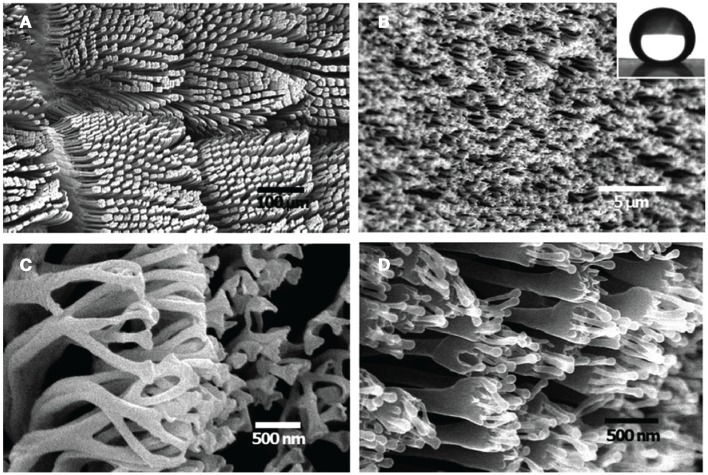
**SEM images of a Tokay Gecko (Gekko gecko) (A,C) and fabricated hierarchical polystyrene (PS) nanohairs with high aspect ratio (AR) (B,D)**. Inset in **(B)** water contact angle of the elongated hierarchical PS nanohairs. From Lee et al. (2012).

The gecko foot adhesion has been shown to rely on van der Waals forces (Autumn et al., 2002). In order to maximize the interfacial contact area, the gecko foot depends on the multiscale structure of the hierarchical keratin foot hairs, which are located on the toe pad of the gecko and are reported to have non-matting and self-cleaning properties (Hansen and Autumn, 2005). Each hierarchical layer allows the gecko foot to adapt and attach to rough and bumpy surfaces by compressing or conforming to varying levels of surface roughness. As is shown in Figure 1, the base layer of the hierarchical structures is composed of soft ridges on the surface of the geckos toes called lamellae which are approximately 1–2 mm in length and are easily compressed for improved contact on rough surfaces. From the surface of the lamellae extend curved hairs or setae which range from 5 to 10 μm wide and from 30 to 130 μm long. The ends of the setae are covered with spatula whose tips are approximately 500 nm long, 10 nm thick, and 200–300 nm wide and are able to conform to small-scale surface roughness. (Bhushan and Sayer, 2007) (Figure 2). The mechanics of attachment and detachment also play a significant role in determining the adhesive performance of the gecko (Autumn et al., 2006b).

Top-down microfabrication techniques have been successfully applied to the creation of highly functional dry adhesives for smooth surfaces (Gorb et al., 2007; Sameoto and Menon, 2010; Song et al., 2013). The present review however provides a summary of the recent progress in the area of nanostructured dry adhesives since 2010. The further challenge of incorporating hierarchical structures involving nanometer-scale components is also given attention. We have excluded carbon nanotube based adhesives, which are in fact very important, but are covered by a recent review (Hu et al., 2013). Similarly we have excluded adhesion models which are discussed in detail in another recent review (Zhou et al., 2013). In particular, we focus on recent reports on fabrication processes.

## Overview of Criteria for Biomimetic Dry Adhesives

In order to mimic the gecko, a dry adhesive should meet the following criteria (Autumn, 2007):

1. adhesion through van der Waals interactions
2. anisotropic adhesion
3. a high pull-off to preload ratio
4. low detachment force when required
5. self-cleaning
6. anti-self-matting/self-adhesion
7. a low to no adhesion state in the absence of shear

Wet pressure-sensitive adhesives conventionally have a Young’s modulus below 100 kPa, whereas geckos achieve similar effective modulus from beta-keratin (Autumn et al., 2006a), which has a much higher bulk modulus in the gigapascal range. The foot hair geometry is essential to achieve this dramatic difference in modulus, and enables the maximization of contact area. As described by Jeong and Suh (2009), four key structural features are considered to be advantageous for modulus translation:

1. High aspect ratio (AR) features
2. Angled/tilted features
3. Multiple length scale hierarchical features
4. Spatula or mushroom cap-terminated features

For practical adhesive applications, additional criteria of importance are: area fill fraction, cost of mold preparation, mold durability, and scalability to large-area production. Also, the actual ability to maintain high adhesion in the presence of contamination, or the ability to easily shed contamination, is crucial for applications such as climbing robots.

### Nanostructure fabrication processes

Outside of CNT-based approaches, which involve directed growth of nanotubes, most practical nanostructured dry adhesive fabrication processes are based on a nanocasting process to form nanowires/nanofibers. The overall process encompasses mold preparation, mold pre-treatment, material infusion, demolding, and post-molding treatments of the demolded adhesive. Each step has important variables that may need to be optimized for a given molding strategy and material system.

**Figure 3:**
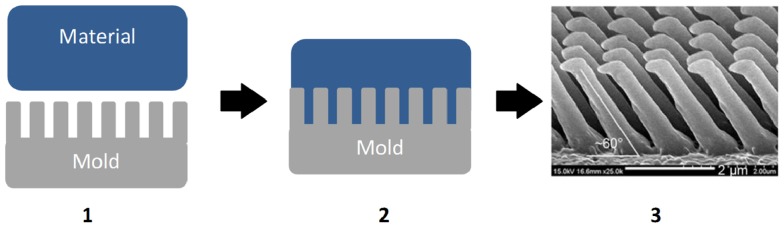
**The nanocasting process for synthetic dry adhesive preparation: (1) mold and material preparation, (2) mold infusion, and (3) demolded dry adhesive**. From Jeong et al. (2009).

A general representation of the nanocasting process is shown in Figure 3. The initial form may either be a positive or negative replica of the desired final product. In the case of a positive replica, an additional negative replication step is then necessary, but this can be advantageous for minimizing damage to the master mold.

**Figure 4:**
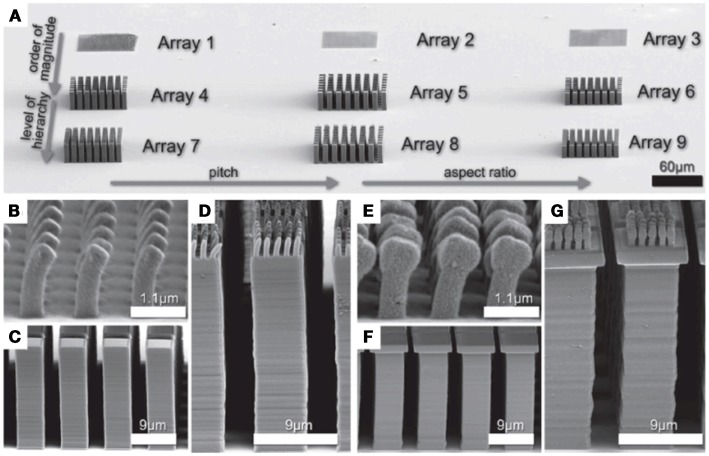
**(A)** Several gecko-inspired arrays fabricated by 3D direct laser writing. **(B)** SEM image of array 1, which contains small single level structures with a width of 500 nm. **(C)** The SEM image shows array 4, which contains pillars of 5 μm width. **(D)** Array 7 consists of the array 1 on top of array 4. **(E–G)** Show corresponding arrays with mushroom-shaped tips. The scale bars refer to the periodicity of the arrays (Röhrig et al., 2012).

An important exception in the recent literature is the application of two-photon lithography to directly write adhesive micro- and nanostructures (Röhrig et al., 2012). This approach allows unprecedented control over the structural configuration of an adhesive (Figure 4), allowing high aspect ratios, tilted, and hierarchical structures, as well as mushroom caps. Presumably, the resulting structures could be transferred into other widely used polymers via a molding process. However, one limitation of two-photon lithography is that the process is sequential and large-area exposures may be very time-consuming.

#### Nanomold preparation

The mold selection or fabrication step is often the most important one. The key structural components of the fibers (AR, tilt, hierarchy, cap) are determined at this stage. Furthermore, the ability to scale up to macroscopic dimensions is important for all but purely fundamental studies, and is largely determined by the mold.

On one hand, many commercially available nanoporous membranes may be used as inexpensive molds, at the expense of limiting the parameter space to the offering of the membrane producer. On the other hand, cleanroom lithographic processes may be expensive and scale poorly.

Several different molding techniques have been used to create nanostructures including melting, UV-crosslinking, polymerization, capillary force nanoimprinting, and two-photopolymerization.

The melting technique involves applying sufficient heat to melt cyclic olefin copolymer (COC), Teflon AF, polypropylene (PP), or low density polyethylene (LDPE) or high density polyethylene (HDPE). In some cases a hydraulic press or injection molding is used to force the melted polymer into the mold cavities formed by membranes, porous anodic aluminum oxide (AAO), silicon nanowires, or molds fabricated by electron beam lithography (EBL) or other technique. A major limitation of using the melting technique is the fabrication or procurement of the molding cavity as commercial injection molding equipment and hydraulic presses are readily available.

Another approach to creating nanostructure arrays is to cast a UV-curable polymer such as soft or hard polyurethane acrylate (PUA) into the mold cavity. UV-curable polymers typically require only a few seconds of exposure to UV light in order to achieve a rapid transition from resin to polymer. Nanostructures have also been fabricated using capillary force nanoimprinting using AAO as a template.

A summary of the nanostructuring techniques is presented in Table 1.

**Table 1:** **Summary of nanostructured dry adhesives in recent literature**.

Structuring technique	Reference	Polymer	Preparation	Hierarchical?	Diameter (nm)	Aspect ratio
Si mold, optical lithography	Jeong et al. (2010a)	h-PUA	UV-crosslink	No	400	5
Si mold, optical lithography	Jeong and Suh (2012)	s-PUA; h-PUA	UV-crosslink	No	700	3.6
3D direct laser writing	Röhrig et al. (2012)	IP-G 780	Two-photon polymerization	No	500, 5600	3–4
				Yes	5600/500	3–4/3–5
Track etch PC mold	Palacio et al. (2013)	PE	Melt	Yes	5000/600	6, 50
Track etch PC mold	Gillies and Fearing (2011)	HDPE; PP	Melt	No	600	33
Track etch PC mold	Lee et al. (2011)	HDPE	Melt	No	300	12, 60
Track etch PC mold	Lee and Bushan (2012)	PP	Melt	No	50, 100, 600, 5000	6, 50, 300, 600
Track etch PC mold	Rodríguez et al. (2013)	PP; PDLLA	Melt	No	200	10
Microsphere lithography on Si	Lee et al. (2011)	LDPE	Melt	No	250–900	1–10
AAO mold	Izadi et al. (2012, 2013)	Teflon AF	Melt	Yes	200	80
AAO mold	Liu et al. (2012)	PI	Polymerization	No	100–200	30–60
AAO mold	Ho et al. (2011)	PC	Capillary force nanoimprinting	Yes	280/90	10/9
					280/110	10/6
				No	280	23
AAO mold	Lee et al. (2012)	PS	Melt	Yes	380/100	2.4, 1.8
					3000/70	10, 7
Ni mold, EBL	Matschuk and Larsen (2013)	COC	Melt	No	40	1–2

### Materials

Whereas microstructured dry adhesives rely on softer materials such as PDMS, with a typical bulk modulus below 5 MPa, nanostructured dry adhesive rely on stiffer materials. A wide variety of polymers have been reported, and there is a natural bias toward easily available and readily processable formulations. The polymers used in the recent literature are summarized in Table 2.

**Table 2:** **Polymers used for nanostructured dry adhesives**.

Polymer	Modulus (GPa)	Reference
IP-G 780	4	Röhrig et al. (2012)
Polyurethane acrylate (PUA), hard	0.32–1.3	Jeong et al. (2010a), Jeong and Suh (2012)
Polyurethane acrylate, soft	0.02	Jeong and Suh (2012)
Polyethylene (PE)	0.4–1.0	Palacio et al. (2013)
Low density polyethylene (LDPE)	0.2	Lee et al. (2011)
High density polyethylene (HDPE)	0.4	Gillies and Fearing (2011), Lee and Fearing (2012)
Polypropylene (PP)	1.3–2	Gillies and Fearing (2011), Rodríguez et al. (2013), Lee and Bushan (2012)
Teflon AF	1.5	Izadi et al. (2013)
Polyimide (PI)	–	Liu et al. (2012)
Polycarbonate (PC)	2.2	Ho et al. (2011)
Polystyrene (PS)	3.2	Lee et al. (2012)
Poly-dl-lactide (PDLLA)	0.3–2.3	Rodríguez et al. (2013)
Cyclic olefin copolymer TOPAS 8007 (COC)	2.6	Matschuk and Larsen (2013)

### Molding techniques

Porous AAO has been used extensively to prepare nanostructured materials since the preparation of ordered pore arrays was reported (Masuda and Fukuda, 1995). It is commercially available as a filtration membrane with a wide range of pore sizes and thicknesses (Whatman Inc.). The laboratory preparation process is also relatively simple, requiring only high purity aluminum foil, a simple electrolyte solution such as dilute sulfuric or oxalic acid, and a DC voltage supply. For membrane fabrication, pore spacing is controlled via the applied potential with self-ordered pore lattices only being obtained with specific voltages and electrolytes. Ordered arrays have been demonstrated with 50–500 nm pitch (Nielsch et al., 2002), with pore diameters typically 50–70% of this value.

The pore arrays are also close-packed in micron-scale domains and this leads to a near ideal filling fraction, which is important both for maximizing adhesive contact area as well as preventing fiber collapse.

Since the pore depth is determined by anodization time, very high and precisely controlled aspect ratio molds can be prepared (AR >1000). The resulting pore diameter may be tuned over a modest range via etching in dilute acids.

**Figure 5:**
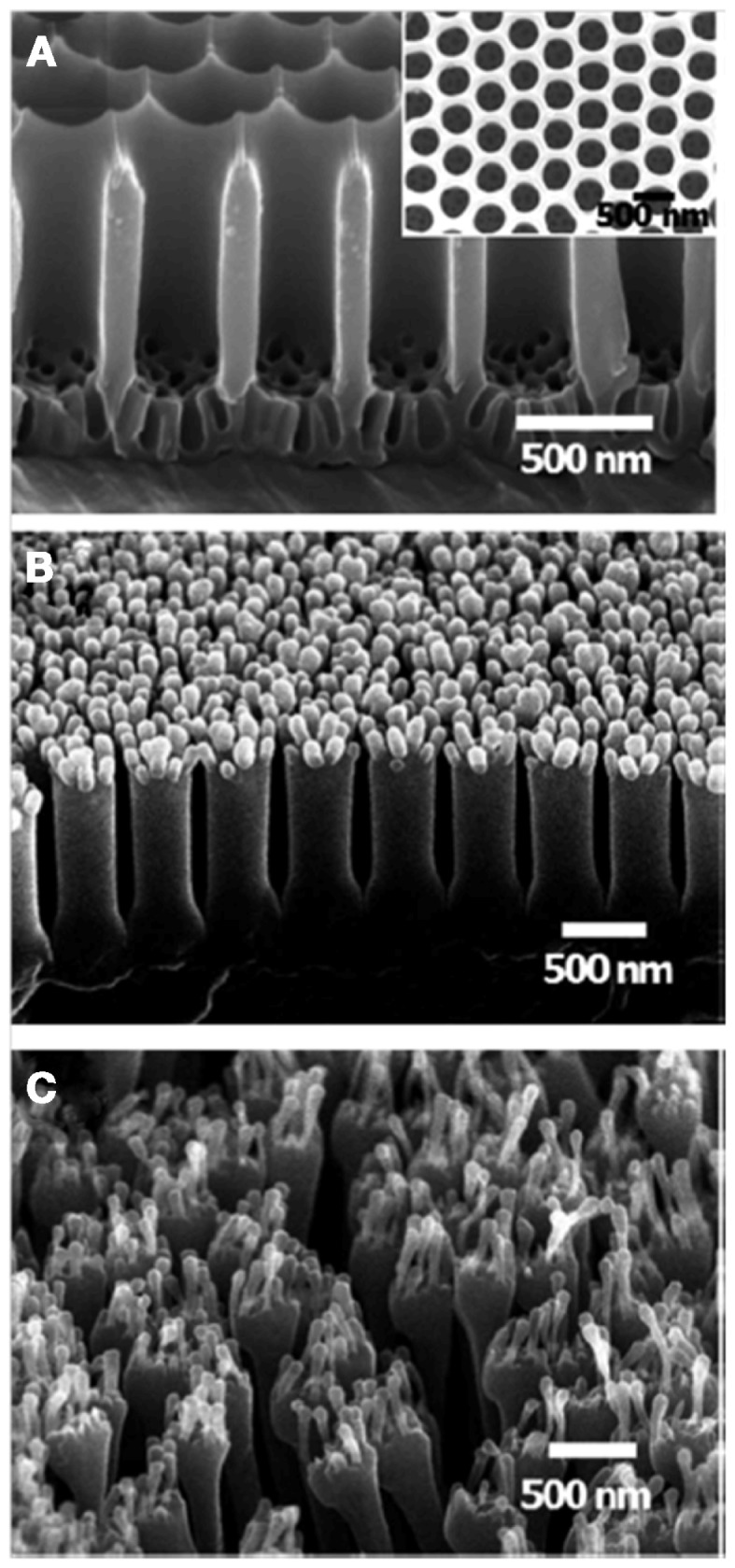
**SEM images of (A) the multi-branched AAO template (inset, top view), (B) the PS nanohairs replicated by removing the template during the wet etching process (denoted DLW), and (C) elongated hierarchical PS nanohairs replicated using the surface-modified template and peel-off process (denoted DL) (tilt angle 45°)**. From Lee et al. (2012).

Furthermore, it has been shown that the anodization voltage may be altered with time to create hierarchical structures (Ho et al., 2011; Izadi et al., 2012; Lee et al., 2012) (Figure 5) and a membrane stacking approach can also be used to obtain a mold with multiple length scales. An extra level of hierarchy has also been introduced through the fingering instability during polymer infusion (Izadi et al., 2013).

As AAO may be etched away readily with dilute acids, a destructive demolding process can be readily effected and, since the mold production and material cost is relatively low, this could be viable for many applications.

Anodic aluminum oxide molds however do not readily lend themselves to tilted structures, but there is an initial report of slanted fibers obtained by changing the angle of release of the template (Lee et al., 2012). Also, precise control of the mold tip shape has not yet been demonstrated.

Even though commercial hydraulic presses and injection molders are readily available, AAO membranes are typically smaller than 50 mm in diameter which places a limitation on the ability to scale up production to large scale sheets to enable nanostructures to become a commercially viable product.

Polycarbonate etch-track membranes are also available commercially as filtration membranes, which has enabled their continued widespread use as a mold for dry adhesives (Gillies and Fearing, 2011; Lee and Bushan, 2012; Lee and Fearing, 2012; Palacio et al., 2013; Rodríguez et al., 2013). The track-etch process, based on selectively dissolving parts of a polymer membrane exposed to nuclear radiation, allows for a very wide range of pore diameters ranging from 50 nm to 12 μm (Millipore, MA, USA), with aspect ratios up to 1000.

Unfortunately, ion tracks are not ordered and the fill fraction of pores is relatively low ( <20%) and higher fill fractions would lead to a broader distribution of pore sizes due to track overlaps. In principle, tilted structures are possible with track-etch membranes, but access to a suitable source of nuclear radiation would be a limiting factor.

**Figure 6:**
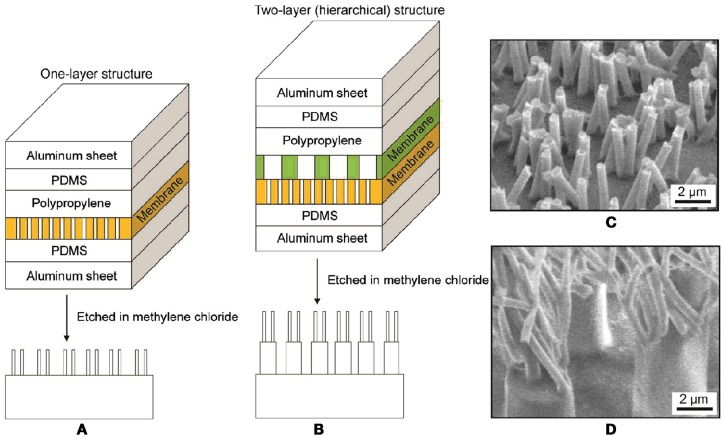
**Sample fabrication processes for (A) one-layer structure and (B) two-layer (hierarchical) structure using one and two membranes in the stack, respectively**. After heating the stacks in an oven, the membranes are etched using methylene chloride to obtain the nanostructured samples. **(C)** SEM image of 600 nm one-layer structure, and **(D)** two-layer structure made from polypropylene. Lee and Bushan (2012).

Due to the large range of diameters, hierarchical micro/ nanomold stacks are possible and have been demonstrated (Lee and Bushan, 2012) (Figure 6). Even though the macroscopic utility of these structures is hampered by the low fill fraction, they still enable local testing of adhesive properties via scanning probe techniques.

Polycarbonate etch-track membranes may currently offer the best choice for large scale production of adhesive nanostructures as a wide range of sheet sizes are available commercially and could relatively easily be paired with hot embossing equipment.

#### Optical lithography

Optical lithography is a conventional microfabrication technique which continues to be exploited in combination with reactive ion etching (RIE) to achieve submicron features. While the technique is limited to the size of the Si wafer, the use of different masks in principle allows the precise control of pitch, diameter, and cross-section.

**Figure 7:**
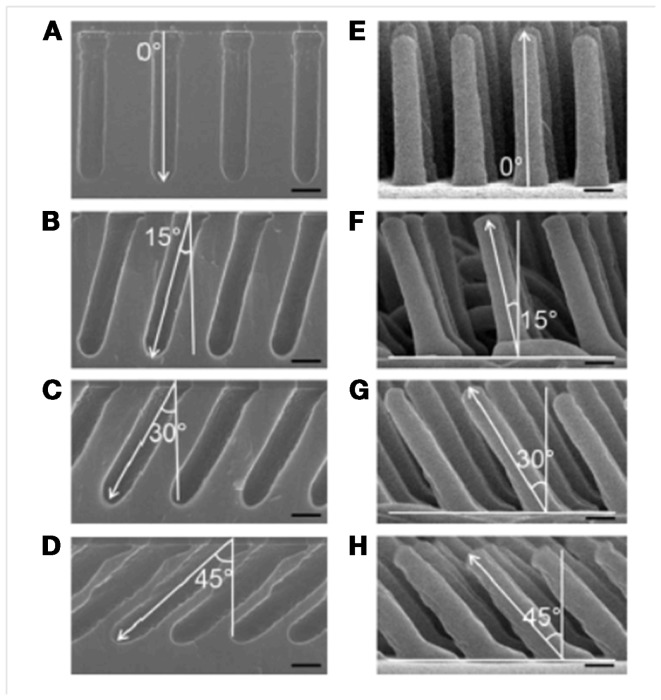
**(A–D)** SEM images of the Si master substrates, prepared via optical lithography and RIE, having angled etch profiles of 0°, 15°, 30°, and 45°, respectively. **(E–H)** SEM images of the h-PUA nanohairs, 2 μm length and 400 nm diameter, replicated from the masters shown in **(A–D)**. Scale bar = 400 nm. From Jeong et al. (2010a).

With the innovation of tilted RIE (Jeong et al., 2009), angled structures are also achievable (Figure 7). Finally, the shape of the tip may be controlled via the RIE process (Jeong et al., 2009) or via post-molding modifications (Jeong and Suh, 2012).

#### Electron beam

Electron beam lithography allows precise control of mold geometry down to the sub-100 nm regime, and this capability may yet allow it to deliver important insights since large areas can be patterned in modern tools. Relatively high aspect ratios are possible in principle because the resist thickness can reach 2000 nm. However, high beam energy is required to maintain small feature diameter and in practice this would reduce the aspect ratio to below 100.

**Figure 8:**
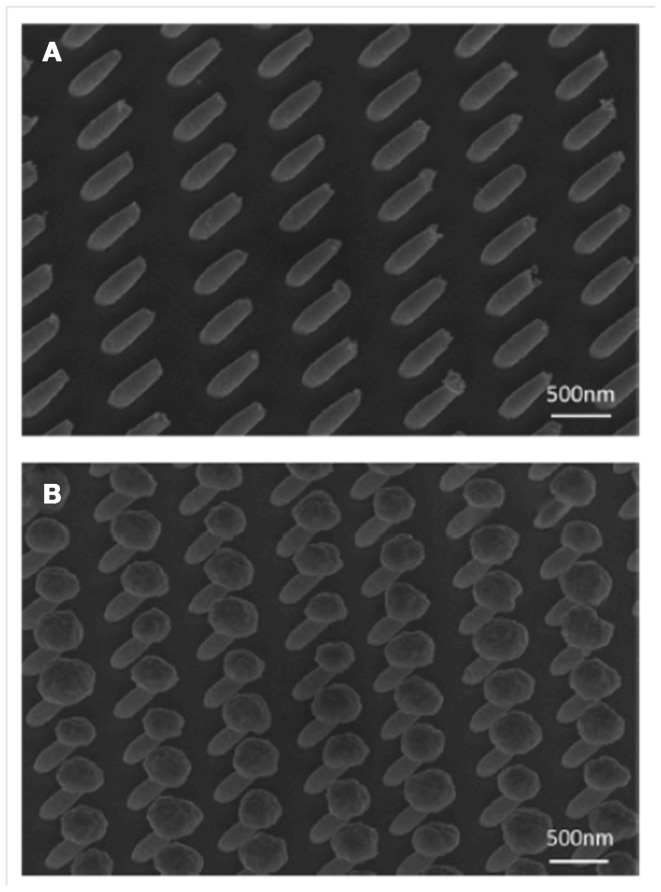
**SEM images of 45° tilted Au pillar arrays by electroplating into tilted hole arrays in electron beam resist**. **(A)** Tilted pillars of 300 nm diameter and 750 nm length without Au over-plating. **(B)** Mushroom-shaped Au pillar array due to over-plating. The images were taken at 45° viewing angle. From Zhang et al. (2012).

Electron beam lithography has also been successfully applied to prepare dry adhesives directly from EBL resists (Tsai et al., 2011) and, furthermore, a nanomold was prepared by overcoating the EBL pattern with Ni (Matschuk and Larsen, 2013). Tilted and capped structures have also been demonstrated in metal structures (Zhang et al., 2012) without conversion to an actual dry adhesive (Figure 8).

#### Microsphere lithography

Microsphere lithography is based on the use of close-packed, micron to submicron colloidal particles that can be transferred to the surface of a target substrate. The pitch of the pattern is determined by the particle diameter where the pattern openings are tuned by RIE of the colloidal particles. Areas as large as 100 mm Si wafers may be coated with a uniform layer.

**Figure 9:**
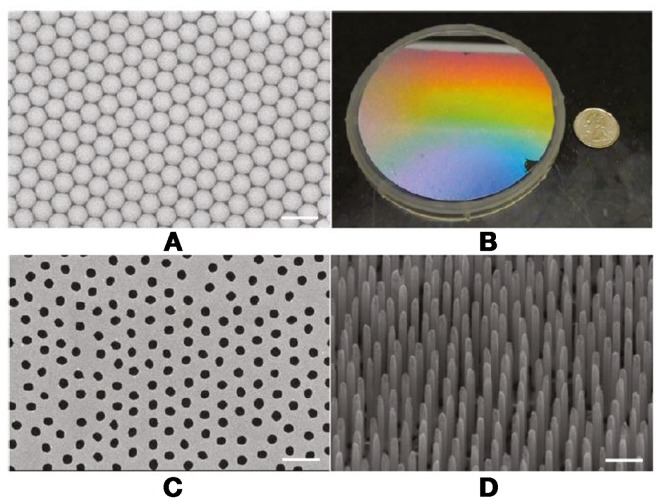
**SEM image of PS microsphere array (A) showing well-ordered structure in microscale**. Optical image of a 4″ wafer **(B)**, showing uniform PS array. Au-coated patterned Si substrate defined by areas not previously covered by the plasma-etched microspheres **(C)**. Slight disordering is induced during the plasma etching process. Au-coated region becomes a catalytic site during metal-assisted electroless etching, which results in well-defined vertical SiNW structures (45° tilt view) **(D)**. Scale bar = 2 μm in all SEM images. From Lee et al. (2011).

A very novel integration of microsphere lithography was presented recently (Lee et al., 2011), where it was combined with metal-catalyzed electroless etching of Si to achieve high AR Si posts, which were then used to create a negative mold for nanocasting (Figure 9).

The fill fraction of the final features is high due to the closely packed nature of the microsphere layer. However, the fill fraction diminishes as the microspheres are etched to tune their diameters. In principle, this could be circumvented through the use of different microsphere diameters.

The aspect ratio achievable through microsphere lithography is entirely dependent on the pattern transfer process and, at present, the highest demonstrated aspect ratio is 10, but it may be possible to extend this further.

Hierarchical structures may be possible with microsphere lithography, if the nanomold is combined with a separately produced micromold based on larger colloidal particles.

In the published implementation, both the Si positive mold and the negative replica are single-use since they are removed by chemical etching. It would be more efficient to avoid these destructive demolding steps, possibly through the use of a mold release layer.

### Mold treatments

#### Surface treatments

The use of mold release agents is common with macroscopic molds, and can be even more critical on the nanoscale, due to the large surface area to volume ratio of the molded features (Matschuk and Larsen, 2013) where the interfacial forces can cause demolding failure and possibly limited reusability of the mold.

For processes using mold surface treatments, the as-fabricated mold often needs a further treatment to ensure proper demolding of the chosen dry adhesive material. The most common strategy is to employ a low-energy release layer, such as a fluoroalkylsilane. This can dramatically improve the nanostructure yield (Matschuk and Larsen, 2013).

#### Mold infusion

The introduction of the target material into a nanomold can be a simple or intricate process depending on the infusing material and the aspect ratio of the mold features. When pre-synthesized polymers are used, the infusion process is driven by a combination of pressure and temperature. Heating above the glass transition temperature of the polymer is required to allow for flow to occur and this process can be relatively time-consuming (up to several hours), which is less attractive for rapid production of large volumes.

*In situ* polymerization and crosslinking is necessary for many materials, such as rubbers (polyurethane, polyimide), which do not melt and cannot be dispersed in a solvent. Also, diffusion of the precursor monomers or oligomers into the mold cavity may be substantially quicker than for pre-synthesized polymers. Conversely, the material properties will depend on polymerization conditions confined on the nanoscale, and may deviate substantially from bulk properties.

Capillary force lithography has been employed to great advantage by Suh and co-workers (Kwak et al., 2010; Jeong and Suh, 2012). This approach doesn’t require high pressures but instead relies on capillary forces in the nanoscale features of the mold to induce infiltration. More recently, capillary force assisted nanoimprinting has been reported (Ho et al., 2011).

#### Demolding

The demolding step is a critical one for nanocasting, due to the high area of interaction between the mold and the replica. The mold may be removed destructively via a solvent (Gillies and Fearing, 2011; Lee and Bushan, 2012; Lee and Fearing, 2012; Palacio et al., 2013; Rodríguez et al., 2013), wet chemical etching (Lee et al., 2011; Izadi et al., 2012; Liu et al., 2012), or by peeling the replica away (Ho et al., 2011; Lee et al., 2012). While the wet etching approach imparts minimal distortion to the replica, it is known that the drying step that follows wet processing leads to clumping of nanofibers (Kustandi et al., 2007).

The peeling approach is of interest since mold fabrication may be expensive and/or time-consuming. Peeling has been used successfully for Si molds (Jeong and Suh, 2012; Jeong et al., 2010a) and is certainly the case for EBL-generated molds, and a detailed study on mold performance and reliability has been carried out (Matschuk and Larsen, 2013).

### Post-molding treatments

**Figure 10:**
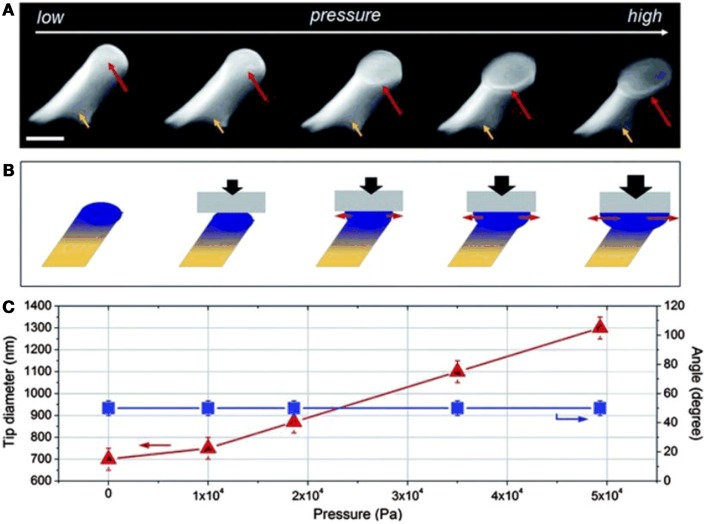
**(A)** Tilted SEM images of angled, PUA nanopillars with different tip shapes from round to increasingly enlarged mushroom- like tips. The tip diameters of nanopillars increased with the increase of applied pressure. Tip diameters of nanopillars, from left to right, are 700, 750, 870, 1100, and 1300 nm, respectively. Length of the nanopillar is 2.5 mm and leaning angle is 50°. **(B)** A schematic illustration showing the tip shape transformation of a nanopillar with gradually increased mechanical loads. **(C)** Plots of tip diameter and leaning angle changes as a function of applied pressure. From Jeong and Suh (2012).

Both structure and surface chemistry of a nanostructured dry adhesive may be modified after demolding. In the first case, some desired shapes such as mushroom caps may be difficult or impossible to demold. A very good example of post-molding treatments involved partially curing PUA (Jeong and Suh, 2012) within a Si mold, then applying pressure to flatten the fiber tips (Figure 10).

In one case, RIE has been used to remove a thin capping layer on molded fibers, used to prevent clumping during solvent removal of the mold (Rodríguez et al., 2013).

One area that is still ripe for further exploration for nanostructured dry adhesives are modifications of the surface chemistry after micro/nanostructuring is complete, such as the use of fluoroalkylsilane to modify the PI surface (Liu et al., 2012). Fluoroalkylsilane or other treatments may allow modification of the surface energy for specific applications, e.g., for enhanced adhesion, resistance to certain chemicals, or self-cleaning properties.

### Measurements on nanoscale dry adhesives

Local nanoscale testing methods are beginning to yield some insight into the adhesion strength of nanoscale structures but it is difficult to compare independent results without a standardized testing method and standardized reporting. Currently, local nanoscale testing is most often being performed with an atomic force microscope (AFM) (Jeong and Suh, 2012; Lee and Bushan, 2012; Lee et al., 2012; Palacio et al., 2013). Macroscopic testing on the other hand has been performed using linear stages and load cells (Gillies and Fearing, 2011; Izadi et al., 2013), universal testing machines (Ho et al., 2011; Rodríguez et al., 2013), a triboindenter (Northern and Turner, 2005), a hanging test (Jeong et al., 2009; Jeong et al., 2010a,b), or pulley system (Lee et al., 2011) with the method used depending on equipment availability and whether normal adhesion, shear adhesion, or a combination of the two is being reported. Macroscopic testing requires careful quantification of actual contact area, especially due to the high stiffness of the bulk polymer supporting the nanostructures.

The effect of tilt angle on adhesion was further confirmed (Jeong et al., 2010b) and it has been shown nanoscale tip shape modifications also enhances adhesion (Jeong and Suh, 2012; Röhrig et al., 2012).

Similarly, the effect of aspect ratio: too high and fibers will collapse, too low and adhesion is also poor and also applies to nanoscale features (Lee et al., 2011; Röhrig et al., 2012).

Adhesive forces in the nanoscale can be very high and material limits may be exceeded (Gillies and Fearing, 2011) indicating that better materials may be needed for improved wear resistance for long term usage.

In at least one material, very high adhesion in nanostructured hierarchical Teflon AF samples may indicate a mechanism other than van der Waals forces, such as contact electrification (Izadi et al., 2012, 2013). Further investigation is needed in order to take advantage of these and possibly other surface effects.

Self-cleaning effects have been tested (Lee and Bushan, 2012; Lee and Fearing, 2012), however general self-cleaning currently remains elusive.

A summary of recent adhesion results are shown in Table 3.

**Table 3:** **Summary of adhesion results**.

Reference	Preload	Tested area	Normal strength	Shear strength
Jeong et al. (2010a)	0.3 N/cm^2^	1 cm^2^	N/A	9.3–38.1 N/cm^2^
Jeong and Suh (2012)	0.3 N/cm^2^	1 cm^2^	N/A	Flat head: 0.32–0.84 N/cm^2^; mushroom head: 1.75–3.10 N/cm^2^
Röhrig et al. (2012)	1.12 N/cm^2^	6.25 × 10^−6^ cm^2^	Hierarchical with mushroom caps: ~0.0184 N/cm^2^; hierarchical with pillars ~0.012 N/cm^2^	N/A
Palacio et al. (2013)	N/A	30 μm diameter spherical tip	136–254 nN	N/A
Gillies and Fearing (2011)	N/A	0.853 mm^2^	N/A	0.03
Lee et al. (2011)	0.1 N/cm^2^	1 cm^2^	N/A	1.1–3.2
Lee and Bushan (2012)	N/A	30 μm diameter spherical tip	98–348 nN	N/A
Rodríguez et al. (2013)	30 mN	1 cm^2^	N/A	PDMS: 0.73 × 10^4^ N/m^2^
				PDLLA: ~0.234 × 10^4^ N/m^2^
				PP: ~2.37 × 10^4^ N/m^2^
Izadi et al. (2012)	5–50 mN	6 mm diameter spherical tip	~1.03–7.35 mN	N/A
Izadi et al. (2013)	5–50 mN	8 mm diameter spherical tip	~0.05–1.60 N/cm^2^	N/A
Liu et al. (2012)	N/A	N/A	66 μN	N/A
Ho et al. (2011)	30 mN	1–7 mm^2^	N/A	Pillars: 4.88 mN; hierarchical: 15.85 mN
Lee et al. (2012)	Normal: 300 nN; shear: 2 μN	26 μm diameter spherical tip	~323–876 nN	~2.68–8.29 μN

## Summary

We have reviewed the state of the art in nanostructured dry adhesive fabrication. Innovative fabrication strategies have yielded key insights and as well as increased adhesive performance. However, there is still much to learn about nanostructured dry adhesives, and what is the best hierarchical implementation for strong, durable, self-cleaning adhesives. Although research seems to be headed in the right direction and nanostructured adhesives with adhesion strength greater than the gecko on some surfaces have been reported, the general dry adhesive for both smooth and porous surfaces is still to be developed, and the gecko continues to provide inspiration in this regard. While climbing robots similar in size to the gecko and capable of climbing on both smooth and rough surfaces has often been cited as the target use for nanostructured dry adhesives other industries that may take advantage of reusable residue-free adhesives are temporary signage and security. With these uses in mind, the ability to fabricate large sheets of nanostructured dry adhesives is desired and may be closest to realization using commercially available membranes and sheets for molds.

## Conflict of Interest Statement

The authors declare that the research was conducted in the absence of any commercial or financial relationships that could be construed as a potential conflict of interest.
